# Association between serum zonulin level and severity of house dust mite allergic asthma

**DOI:** 10.1186/s13223-021-00586-7

**Published:** 2021-08-31

**Authors:** Shereen A. Baioumy, Aya Elgendy, Shereen M. Ibrahim, Sara I. Taha, Shaimaa H. Fouad

**Affiliations:** 1 Department of Microbiology and Immunology, Faculty of Medicine, Zagazig University, Zagazig, Egypt; 2 Department of Internal Medicine/Allergy and Clinical Immunology, Faculty of Medicine, Ain Shams University, Abbasia, Cairo, Egypt; 3 Department of Parasitology, Faculty of Medicine, Zagazig University, Zagazig, Egypt; 4 Department of Clinical Pathology, Faculty of Medicine, Ain Shams University, Cairo, Egypt

**Keywords:** Asthma, Zonulin, Asthma grade, Severity, House dust mites, Intestinal barrier

## Abstract

**Background:**

Increased intestinal permeability, either due to the exposure to antigens in asthmatic patients or due to a barrier defect, plays a critical role in susceptibility to environmental allergens. House dust mite allergy occurs more commonly than any other type of allergy among Egyptian asthmatic patients.

**Aim:**

To assess the relation between serum zonulin level as a marker of increased intestinal permeability and the severity of house dust mite allergic asthma.

**Methods:**

A case–control study which included 48 patients with house dust mite allergic asthma and 48 healthy control subjects attending the Allergy and Immunology Unit, Microbiology and Immunology Department, Faculty of Medicine, Zagazig University.

**Results:**

A statistically significant difference was detected between the two studied groups with respect to serum IgE and serum zonulin levels (p ˂ 0.001 and ˂ 0.001, respectively). The mean serum zonulin was equal to 258.3 ± 153.01 ng/ml in the asthmatic group and 80 ± 13 ng/ml in the control group. Serum zonulin level significantly increased with the increase of asthma severity (p ˂ 0.001). The cut off value of serum zonulin was ≥ 198 ng/ml, and the area under the curve was 0.76. It displayed sensitivity equal to 80% and specificity equal to 71.4%. Its negative predictive value was equal to 83.3%.

**Conclusion:**

Intestinal barrier dysfunction contributes to the pathogenesis of allergic asthma. Serum zonulin level reflects an increase in intestinal permeability. Zonulin acts as prognostic factor of severity in asthma. Correction of the gut barrier defect may have a potential positive prognostic effect in asthma.

## Introduction

Asthma is a common chronic airway disease characterized by airway inflammation, hyper-responsiveness and variable airway obstruction, which is often attributed to gene–environment interactions [1].

Epidemiologic studies have shown that sensitization to indoor allergens is an important risk factor for the occurrence of acute attacks of asthma [2].

The most prevalent indoor allergens include house dust mites (HDMs), animal dander, moulds and cockroaches. Of these, HDMs, especially *D. pteronyssinus* and *D. farinae*, are considered the major perennial indoor allergen sources inducing allergic sensitization worldwide [3].

Environmental factors, microbiome, epithelial cells and immune cells show a dynamic cross talk at the skin and mucosal barriers in the development of atopic dermatitis, allergic rhinitis, eosinophilic esophagitis and asthma [4].

Recent trends in targeting allergic disorders are directed towards personalized medicine approach which necessitates novel developments in the area of disease phenotyping and endotyping, as well as the development and application of reliable biomarkers [4].

Increased intestinal permeability due to the exposure to antigens in asthmatic patients may play a role in susceptibility to environmental allergens. Therefore, correction of the gut barrier defect may be an additional novel approach for asthma treatment [5].

More than 50 proteins act together to create effective tight junctions which are crucial to having an efficient gut mucosal barrier. A 47 kDa protein named zonulin enhances and reversibly alters intestinal permeability [6, 7]. It also shares in innate intestinal immune response. An increase in serum zonulin level has been reported in many autoimmune diseases in which a defective intestinal barrier plays a role in their pathogenesis [8]. Chronic inflammation due to environmental stimuli results in up-regulation of serum zonulin. An increase in serum zonulin was noticed to be significantly correlated to disease severity in atopic dermatitis patients [9].

## Patients and methods

This is a case control study which includes 96 subjects who attended the Allergy and Immunology Unit, Microbiology and Immunology Department, Faculty of Medicine, Zagazig University.

### Sample size

Assuming that serum zonulin level in case group versus control group is (11.3 ± 3.7 ng/ml) and (9.3 ± 3.3 ng/ml) respectively with confidence level 95% and power 80%, the sample size is 96 (48 in every group).

They are divided into two groups.

*Group 1 (Control group)* includes 48 healthy subjects who fulfill the same exclusion criteria as the patients, and have never had asthma or eczema. They were health workers recruited from the hospital staff members.

*Group 2 (Asthmatic patients)* includes 48 adult (≥ 18 years old) subjects with allergic asthma according to Global Initiative for Asthma consensus report (GINA), 2020 [10].

### Inclusion criteria

Allergic asthma patients were selected as having:

- Obstructive pattern; Low FEV1/FVC on pulmonary function test;
- A positive skin prick test for house dust mites.

### Exclusion criteria

1. Evidence of digestive disease;
2. Any other condition associated with increased intestinal permeability, including cystic fibrosis, intestinal parasitic infestation, inflammatory bowel diseases;
3. Food allergy or a positive skin test against common food allergens including, fish and shrimps;
4. Patient refusal to participate.

Each patient was subjected to the following:

1. Full detailed history and examination to exclude any comorbid condition that may affect the result of the study;
2. Full lab to exclude other causes that may increase the intestinal permeability, including liver function test, anti-tissue transglutaminase, stool analysis, stool culture, rheumatoid factor, Hb A1c, ESR, CBC with differential count and fecal calprotectin;
3. Full detailed allergic history and clinical examination of the respiratory system.
4. Chest x-ray to exclude other lung pathology;
5. Pulmonary function tests with special emphasis on FEV1and FEV1/FVC using SPIROMETRICS;
6. Total serum IgE for each patient using enzyme-linked immunosorbent assay (ELISA).
7. Skin prick testing to common allergens;
8. Asthma severity score, calculated according to GINA, 2020 [10].
9. Serum zonulin level detection by ELISA.

### Asthma severity scoring

Asthma severity was classified into grades I–IV (I being the mildest and IV the most severe). Grading was based on events that occurred over the past 6 months, including day and night symptoms, effect of asthma on daily activity, use of steroids and peak expiratory flow rate as defined by GINA guidelines 2020 [10]. Pulmonary function tests were performed for all patients using a fully computerized Spirometer (Jaeger MasterScreen™ IOS, version 5.2 manufactured by VIASYS Healthcare GmbH, Hoechberg, Germany). Pulmonary functions were assessed using forced expiratory volume in 1 s (FEV1), forced vital capacity (FVC) and the FEV1/FVC ratio, measured and expressed as a percentage of predicted values, with a ratio higher than 0.8 being normal.

### Skin testing

- Skin testing was performed according to Bernstein et al. [11].
- Allergen extracts of skin testing were standardized allergen extracts provided by Hamilton (Omega, Allergy OVERSEAS consultant Inc., Canada). The test was performed using the following allergens: for aeroallergens: (house dust mites: *Dermatophagoides* (D.) *pteronyssinus* and *Dermatophagoides* (D.) *farnae* mites, grass, mixed pollens, mixed molds, tobacco, cotton, wool, cockroach and hay dust) and for food allergens: (egg, fish, shrimps, peanuts and cow milk).
- Histamine dihydrochloride (10 mg/ml) was used as a positive control, while saline was used as a negative control.
- Subjects were asked to stop antihistamines a week before skin testing.
- The largest diameter of the wheal was measured, and it was considered positive if it was ≥ 3 mm [12].
- Those with positive tests for food allergy were excluded from the study.

### Sample collection

Five millilitre blood samples were collected by venipuncture under complete aseptic conditions. Samples were left to clot then centrifuged at 1000×*g* for 15 min. Sera were collected and stored at − 20 °C. Sera were used to measure serum levels of total IgE, and zonulin.

### Serum level of total IgE

Quantitative measurement of serum level of total IgE was done using a commercially available sandwich enzyme-linked immunosorbent assay (ELISA) Kit supplied by Chemux Bioscience, Inc. (CA, USA, Cat No. 10602) according to the manufacturer’s instructions. The results were expressed in IU/ml. The total IgE level in a normal allergy-free adult is less than 150 IU/ml of serum. The minimum detectable concentration of IgE by this assay is estimated to be 5.0 IU/ml. The Absorbance of standards and samples were measured at 450 nm using a microtiter plate ELISA reader (Biotek, USA).

### Measurement of serum zonulin level

Quantitative measurement of serum level of zonulin was done using a commercially available sandwich enzyme-linked immunosorbent assay (ELISA) Kit supplied by MyBiosource, Inc. (USA, Cat No: MBS774026) according to the manufacturer’s instructions. The results were expressed in ng/ml. Absorbance of standards and samples were measured at 450 nm using a microtiter plate ELISA reader (Biotek, USA). The kit detection range was 20–800 ng/ml.

### Statistical analysis

The collected data were processed and coded before being analyzed using SPSS program version 23. Quantitative data were presented as minimum, maximum, mean and SD.

Qualitative data were presented as count and percentage. Student t test was used to compare quantitative data between two groups and One Way ANOVA test was used to compare more than two groups. Pearson’s correlation was used to measure linear relationship between two continuous variables. ROC curve was used to measure the diagnostic validity of quantitative variables and linear regression test was used to measure the independent effect of some factors on quantitative outcome variable. p value < 0.05 was considered statistically significant (Fig. 2).

## Results

The current study is a case–control study which includes 96 subjects who attended the Allergy and Immunology Unit, Microbiology and Immunology Department, Faculty of Medicine, Zagazig University during the year 2021.They were divided into two groups, group 1 included healthy controls with a median age of 28 [n = 48] and group 2 included patients with allergic asthma according to Global Initiative for asthma consensus report (GINA), 2020 with a median age of 30 [n = 48].

Regarding the asthmatic patients, the mean value of age is 30.67 ± 15.609, and most of the patients are female (62.5%) the majority of whom live in rural areas (54.16%). 39 patients (81.3%) have positive family history of atopy. All patients displayed positive skin prick test results to house dust mites, with most of them (70.8%) sensitive to *D. pteronyssinus*, while 60.4% sensitive to *D. farina*. 14 patients were mono sensitized to a single type of house dust mites and 34 patients were polysensitized to both types. As for the grade of asthma severity, 4 patients (8.3%) had grade 1 severity, 24 patients (50%) had grade 2 asthma severity, 12 (25%) patients had grade 3 severity, and 8 patients (16.7%) had grade 4 asthma severity (Table 1).

**Table 1:** Description of studied subjects

Variable	Control group (n = 48)	Percentage %	Asthmatic patients (n = 48)	Percentage %
Sex				
Male	28	85.3%	18	37.5
Female	20	14.7%	30	62.5
Age (years)				
X ± SD	32.7 ± 11.2		30.67 ± 15.609	
Median	28		30	
Residence				
Rural	34	70.8%	26	54.16
Urban	14	29.2%	22	45.84
Family history of atopy				
Positive	11	22.9%	39	81.3
Negative	37	77%	9	18.8
Skin prick				
Negative	48	100%	0	0
Positive	0	0%	48	100
House dust mites			48	100
* D. pteronyssinus*	–	–	34	70.8
* D. farina*			29	60.4
Both			34	70.8
Asthma grade	Asthma grade 1		4	8.3
	Asthma grade 2		24	50.0
	Asthma grade 3		12	25.0
	Asthma grade 4		8	16.7

Concerning the control group, the mean value of age was 32.7 ± 11.2. Most patients were males (85.3%) and 70.8% of them lived in rural areas. As for the family history of atopy, only 11 (22.9%) patients had positive family history of atopy and all of them displayed negative skin prick test results.

Upon comparing serum total immunoglobulin E (IgE) level between asthmatic patients and control subjects, there was a highly significant statistical difference between both groups (p = 0.000). Asthmatic patients displayed a higher statistically significant level of serum total IgE. The mean serum IgE was equal to 233.3 ± 103.4 IU/ml in the asthmatic group and 84.4 ± 18.6 IU/ml in the control group (Table 2).

**Table 2:** Comparison between total IgE levels and zonulin levels among both control and case groups

Variable	ControlGroup 1 (n = 48)	Asthmatic patientsGroup 2 (n = 48)	F	P
Total IgE level (IU/ml)				
Mean ± SD	84.4 ± 18.6	223.3 ± 103.4	4.8	0.000HS
Zounlin (ng/ml)				
Mean ± SD	80 ± 13	258.3 ± 153.01	6.12	0.000HS

NS, Non-significant; S, Significant; HS, Highly significant

Similarly, there was a highly significant statistical difference between both groups as regards serum zonulin level. Asthmatic patients displayed a higher statistically significant level of serum zonulin than control subjects. The mean serum zonulin was equal to 258.3 ± 153.01 ng/ml in the asthmatic group and 80 ± 13 ng/ml in the control group (Table 2).

Factors affecting serum total IgE level were male sex, asthma grade, and positive skin prick test. Male patients displayed a statistically significant higher serum total IgE level (p = 0.03). The mean value of serum total IgE was 272.89 ± 141.33 among male patients and 193.53 ± 56.38 IU/ml among female patients. Besides, post hoc test showed a significant difference between grade 1 vs grade 2 as regards serum total IgE. As the asthma severity increased, there was a significant corresponding increase in the serum total IgE (p = 0.02). Moreover, there was a highly significant statistical difference as regards the mean value of serum total IgE between patients displaying positive skin prick test results and those displaying negative skin prick test results (p = 0.002). The mean value of serum total IgE among patients displaying positive skin prick test results was 244.41 ± 114.85 IU/ml (Table 3).

**Table 3:** Factors affecting total IgE level

Variables	Total IgE		Test value	p value
	Mean	SD		
Sex				
Male	272.89	141.33	2.29*	0.03 S
Female	193.53	56.38		
Residence				
Urban	219.27	93.30	0.25*	0.81 NS
Rural	226.69	112.99		
Age (years)				
< 30	240.00	120.07	1.12*	0.27 NS
> 30	206.58	82.78		
Asthma grade				
Asthma grade 1	154.50	.58	5.77**	0.02 S
Asthma grade 2	205.87	88.23		
Asthma grade 3	241.08	113.93		
Asthma grade 4	283.25	132.03		
Skin prick				
Negative	172.00	34.37	3.33*	0.002 HS
Positive	244.41	114.85		

*Student t test

**One Way ANOVA test (post hoc test shows significant difference between grade 1 vs grade 2)

NS, Non-significant; S, Significant; HS, Highly significant

**Fig. 1:**
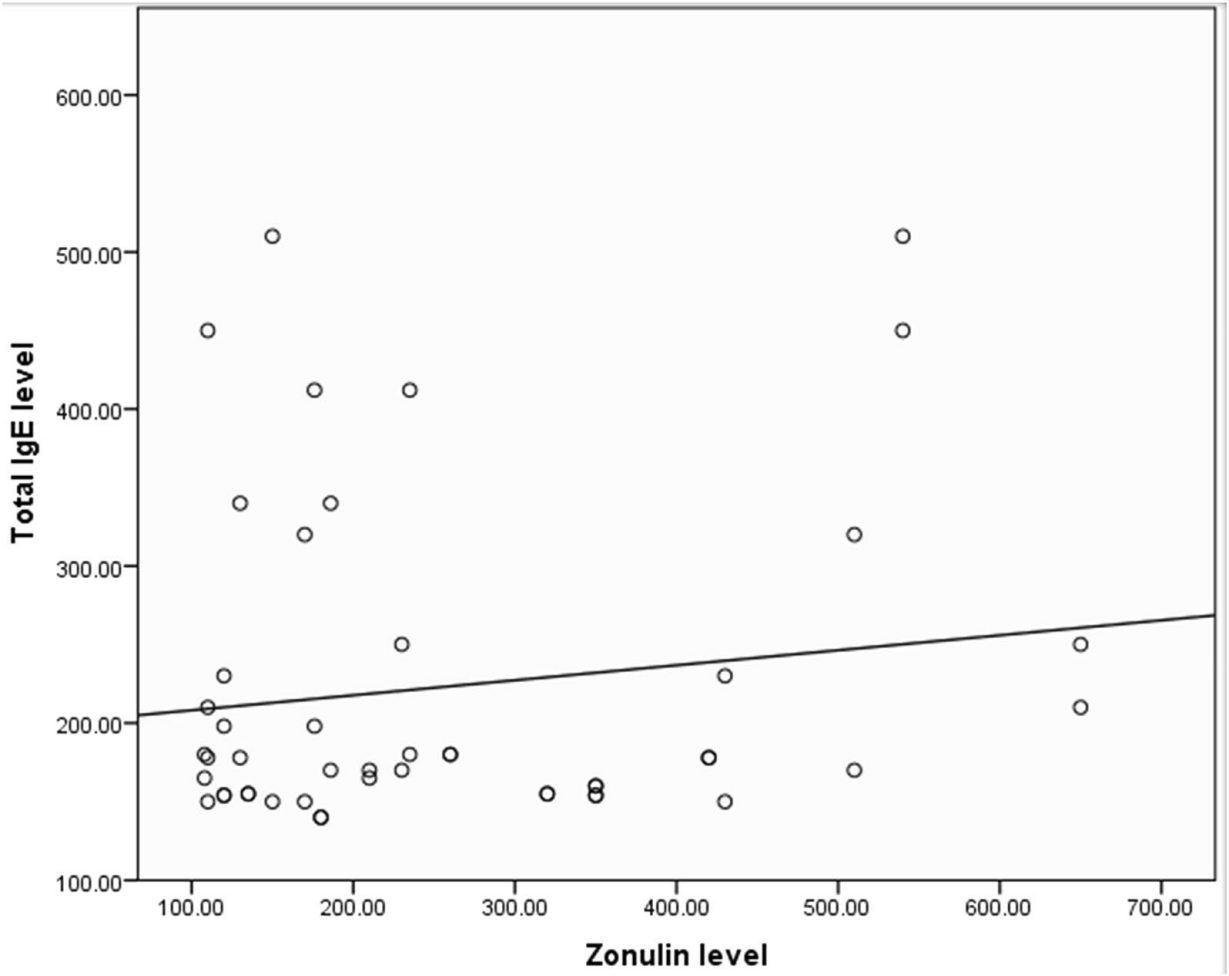
Correlation between serum zonulin level and total serum IgE level

With regard to the correlation between serum zonulin level and serum total IgE, it did not reach a level of statistical significance (p = 0.34) (Table 4 and Fig. 1)

**Table 4:** Factors affecting zonulin level

	Zonulin level		Test value	p value
	Mean	SD		
Sex				
Male	290.11	163.89	1.12*	0.27 NS
Female	239.27	145.59		
Residence				
Urban	315.55	157.34	2.51*	0.02 S
Rural	209.92	133.81		
Age (years)				
< 30	261.67	152.41	0.15*	0.88 NS
> 30	255.00	156.83		
Asthma grade				
Asthma grade 1	127.50	8.66	64.54**	< 0.001 HS
Asthma grade 2	209.50	103.91		
Asthma grade 3	218.50	75.50		
Asthma grade 4	530.00	87.83		
Skin prick				
Negative	299.29	182.05	1.20*	0.24 NS
Positive	241.47	138.88		
	Zonulin level			
Total IgE				
Pearson correlation	0.14			
p value	0.34NS			

*Student t test

**One Way ANOVA test (post hoc test shows significant difference between grade 1 vs grade 2, grade 1 vs grade 3, grade 1 vs grade 4, grade 2 vs grade 4 and grade 3 vs grade 4)

NS, Non-significant; S, Significant; HS, Highly significant

**Fig. 2:**
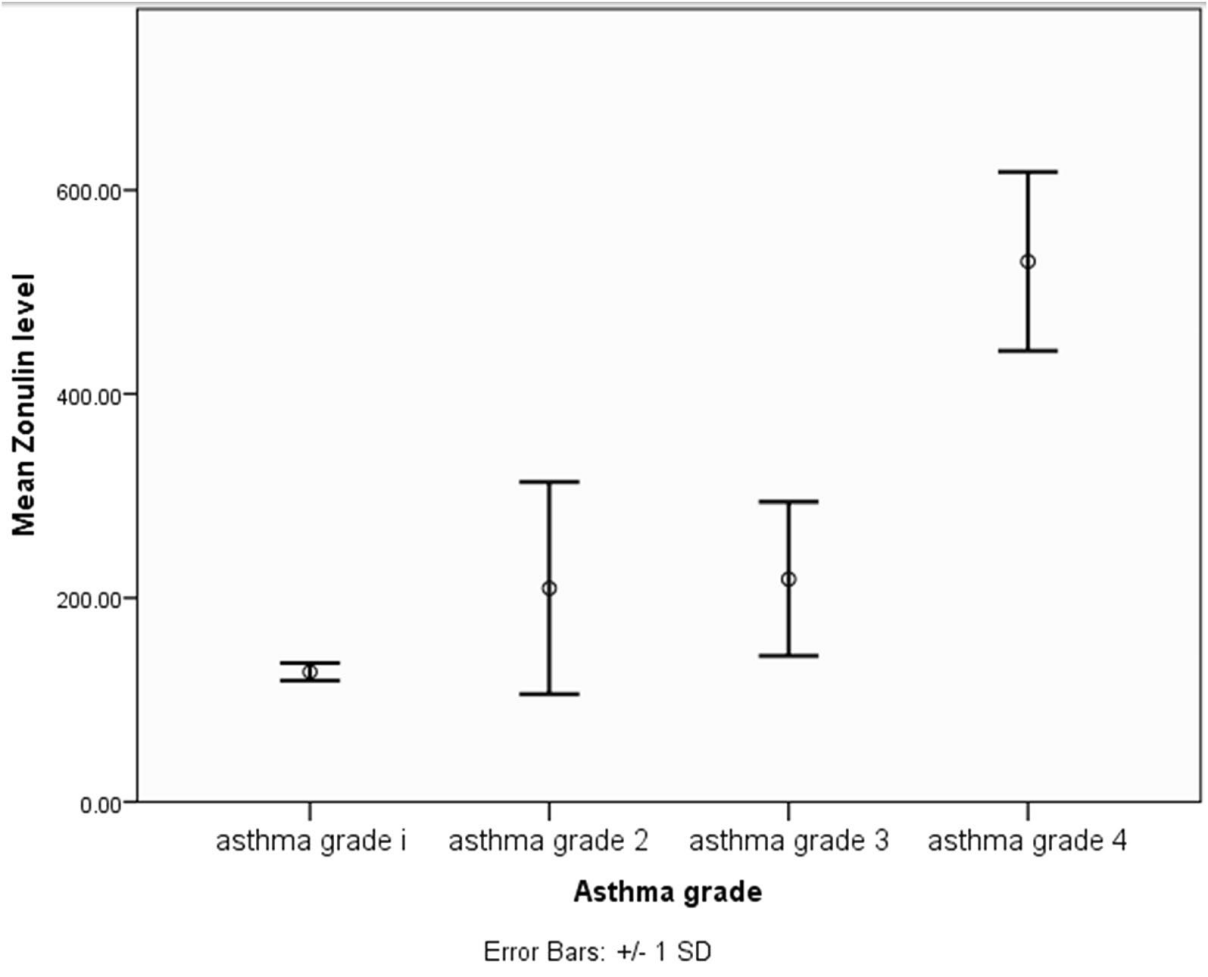
Correlation between asthma grade of severity and serum zonulin level

As for the factors affecting serum zonulin level, they were (the place of) residence and asthma grade. There was a statistically significant difference in the serum zonulin level between patients living in urban areas and those living in rural areas (p = 0.002). The mean value of serum zonulin was significantly higher in patients living in urban areas as it equaled 315.55 ± 157.34 ng/ml. Post hoc test showed a highly significant statistical difference between grade 1 vs grade 2, grade 1 vs grade 3, grade 1 vs grade 4, grade 2 vs grade 4 and grade 3 vs grade 4 as regards serum zonulin level (p ˂ 0.001) (Table 4 and Fig. 2)

Grade of asthma had an independent significant effect on zonulin level (p < 0.001), while serum total IgE had no significant effect on zonulin level via linear regression analysis (p = 0.684 NS) (Table 5).

**Table 5:** Linear regression analysis for factors affecting zonulin level

	Unstandardized coefficients		Standardized coefficients	Sig	95.0% confidence interval for B	
	B	Std. error	Beta		Lower bound	Upper bound
Grade 3 or 4	167.317	29.962	0.838	< 0.001 HS	107.007	227.628
Total IgE	0.075	0.183	0.062	0.684 NS	− 0.293	0.443

NS, Non-significant; S, Significant; HS, Highly significant

**Fig. 3:**
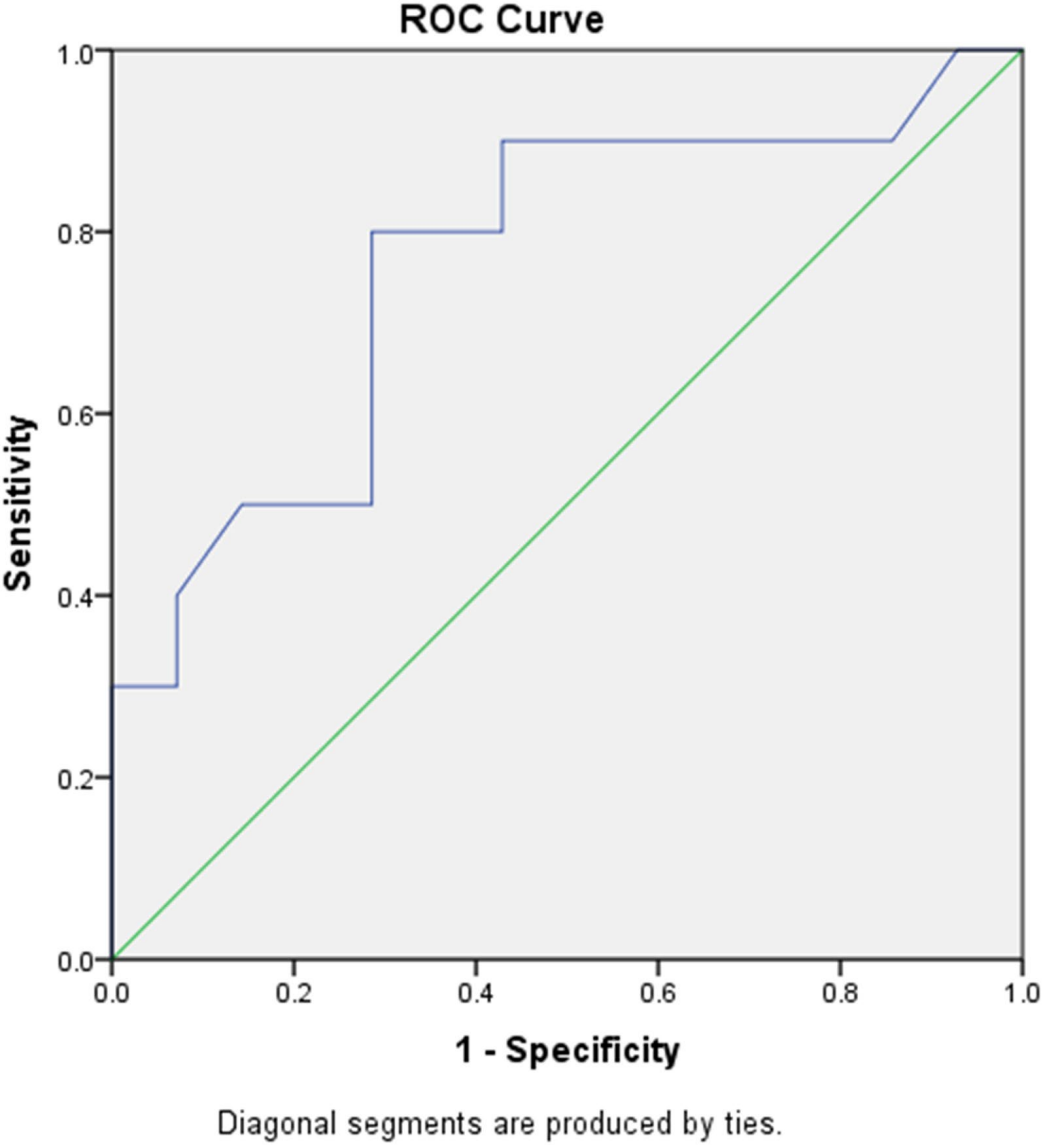
Roc curve of zonulin level for differentiation between (grade 1 or 2) vs (grade 3 or 4)

Roc curve was done to assess the validity of zonulin level for differentiation between (grade 1 or 2) vs (grade 3 or 4) (p = 0.002). It displayed sensitivity equal to 80% and specificity equal to 71.4%. Its positive predictive value was equal to 66.7% and negative predictive value was equal to 83.3%. The cut off value of serum zonulin was ≥ 198 ng/ml. The area under the curve was 0.76 (Table 6 and Fig. 3)

**Table 6:** Validity of zonulin level for differentiation between (grade 1 or 2) vs (grade 3 or 4)

Parameters	Cut off point	AUC	Sig	Sensitivity (%)	Specificity (%)	+PV (%)	−PV (%)	95% confidence interval	
								Lower bound	Upper bound
Zonulin	≥ 198	0.764	0.002 HS	80	71.4	66.7	83.3	0.622	0.906

## Discussion

Asthma is a chronic inflammatory lung disease which is characterized by airway inflammation, intermittent airflow obstruction, and bronchial hyper-responsiveness [13]. Allergic asthma can be sparked by allergen sensitization in which house dust mites (HDM) are among the most prevalent indoor allergens [14]. Exposure to dust mite allergens is a risk factor for clinical asthma in sensitized subjects [15].

A detailed clinical history and physical examination followed by the detection of total serum IgE level and IgE immunoreactivity against specific allergens still represents the cornerstone in approaching allergic disorders [4].

The relationship between allergic asthma and intestinal permeability is a subject of research interest. An increase in the intestinal permeability may result in facilitating the entry of allergenic proteins from the intestinal lumen into the systemic circulation, thereby causing activation of the adaptive immune system, allergen sensitization and/or extra-intestinal inflammation [16].

In the current study the mean value of age is 30.67 ± 15.609, and most of the patients are female (62.5%) the majority of whom live in rural areas (54.16%). 39 patients (81.3%) have positive family history of atopy. All patients displayed positive skin prick test results to house dust mites, with most of them (70.8%) sensitive to *D. pteronyssinus*, while 60.4% sensitive to *D. farina*. 14 patients were mono sensitized to a single type of house dust mites and 34 patients were polysensitized to both types. As for the grade of asthma severity, 4 patients (8.3%) had grade 1 severity, 24 patients (50%) had grade 2 asthma severity, 12 (25%) patients had grade 3 severity, and 8 patients (16.7%) had grade 4 asthma severity. Asthma patients were more commonly living in rural areas (54% rural vs 45.84% urban asthmatics) and all of them had positive skin prick test response to house dust mites, DP and Df (70% and 60%, respectively). When serum total immunoglobulin E (IgE) level was compared in asthmatic patients and control subjects in the present study, it was found that asthmatic patients displayed a higher statistically significant level of serum total IgE. It was statistically significantly higher among male asthmatic patients. There was a positive correlation between total serum IgE level and asthma severity grade and skin prick test results.

The disparity in the development of total IgE between males and females is the consequence of the fact that the levels of total IgE depend on many other factors, such as parasitic infestations, smoking, pollution, local diet and different genetic background in which males are at greater risk of exposure [17].

The impairment of the epithelial barrier is a corner stone in the development of allergic diseases. Increased intestinal permeability, either due to the exposure to antigens in asthmatic patients or due to a barrier defect, play a critical role in susceptibility to environmental allergens [18]. The process responsible for enhanced intestinal permeability in asthma is still indeterminate. There is an association between enhanced intestinal permeability and the severity of the asthma, which suggests that it is a bronchus to gut disease [19]. This could be attributed to the fact that mucosal defects can exist simultaneously in many organs and antigenic stimulation as well as environmental factors can result in its clinical expression in a single organ [20]. For instance, identical histological mucosal changes have been observed in both duodenal and bronchial mucosa simultaneously [8].

Therefore, correction of the gastrointestinal barrier defect is possibly an additional alternative approach for controlling asthma severity [5–21] as it leads to reduction of epithelial liability to damage and resultant inflammatory and remodeling reaction. In addition to growth factors, several peptides, such as AT-1001, a peptide inhibitor of zonulin, can repair barrier function [22]. It is, therefore, essential to study serum zonulin level and its regulation in patients with asthma.

The present study revealed that asthmatic patients displayed a higher statistically significant level of serum zonulin than control subjects. Patients living in rural areas had a significantly higher level on serum zonulin. Furthermore, there was a significant correlation between zonulin level and asthma grade. The higher the zonulin level, the greater the grade of asthma severity. Nevertheless, we found that there was no difference in serum levels of zonulin subjects regarding gender and age. The cut off value of serum zonulin level to differentiate between grade 1–2 asthma severity and grade 3–4 asthma severity was ≥ 198 ng/ml. In addition, serum zonulin showed a sensitivity equal to 80%, a specificity equal to 71.4%, a positive predictive value equal to 66.7% and a negative predictive value equal to 83.3%. There was no statistically significant correlation to serum total IgE concentration. Grade of asthma had an independent significant effect on zonulin level while serum total IgE had no significant effect on zonulin level via linear regression analysis.

A study conducted by Benard and colleagues to assess the intestinal permeability in asthmatics via administering oral radioactive CrEDTA and estimating its urinary recovery revealed that asthmatic patients had increased intestinal permeability in comparison. Contrary to the current results, it was found not to be correlated to the severity of asthma as deduced by pulmonary function test measurements or to the use of steroid treatment [23]. This may be due to the difference in the method used to assess intestinal permeability.

Furthermore, a study performed by Cervantes-García and collaborators aiming at assessing the outcome of oral *Lactococcus lactis* NZ9000 use on airway inflammation and lung remodeling in asthmatic rats and its relation to the preservation of the intestinal barrier, concluded that oral *L. lactis* could be used for asthma prevention through its maintenance of an adequately functioning intestinal barrier [24].

Initial findings by Fasano A imply that a group of asthmatic patients has both high levels of serum zonulin and enhanced intestinal permeability [25]. These findings indicate that antigens presentation and subsequent lung inflammation occur as result of the passage of antigens through lung and intestinal mucosa and submucosa and the subsequent stimulation of the immune system resulting in lung inflammation [26].

Moreover, a study performed by Yamaide et al. 2020 to examine the differences between serum zonulin level among allergic children and non-allergic children concluded that serum zonulin was significantly greater in children with allergy (Food allergy, Bronchial Asthma). In addition, it was significantly higher in patients with food allergy than in bronchial asthma patients. However, their results are not completely reliable as food allergy can be the main reason for increased intestinal permeability and this explains the higher increase in zonulin level among food allergy patients than among asthmatic ones [18].

Up to the present moment, no studies have been conducted to assess the relationship between serum zonulin level and asthma severity or to assess its correlation to different residential distribution. A study was performed by Sheen et al. to assess serum zonulin level in atopic dermatitis (AD) and its correlation to disease severity. They found that AD group had a greater median serum zonulin level than the control group and that serum zonulin level had significantly positive correlations with age and the SCORAD index, but not with total IgE, total eosinophil count (TEC), or the number of allergens to which a child is sensitized. They concluded that each 1 ng/ml increase in serum zonulin was associated with a 15% greater risk of moderate–severe AD. [9] Their results agree with the current results as regards the correlation between serum zonulin level and total serum IgE level. Both revealed no statistically significant correlation.

The current study did not assess whether the increased permeability was the cause or the consequence of bronchial asthma. It disclosed the significant positive correlation between zonulin level and asthma severity. Hence, this suggests that increased intestinal permeability has a deleterious impact on asthma severity. Further studies are needed to assess its role in the pathogenesis of asthma.

## Conclusion

Intestinal barrier dysfunction contributes to the pathogenesis of allergic asthma. Serum zonulin level reflects an increase in intestinal permeability. Zonulin acts as prognostic factor of severity in asthma. Correction of the gut barrier defect may have a potential positive prognostic effect in asthma.

## Data Availability

All the data needed to support the current findings could be found in a supporting sheet.

## Abbreviations

AD: Atopic dermatitis
ANOVA test: Analysis of variance test
CBC: Complete blood count
CrEDTA: Chromium ethylenediaminetetraacetic acid
COX: Cyclooxygenase
Ig E: Immunogloulin E
D: Dermatophagoides
Df: Dermatophagoides farnae
DP: Dermatophagoides pteronyssinus
ELISA: Enzyme-linked immunosorbent assay
ERs: Estrogen receptors
ESR: Erythrocyte sedimentation rate
Fev1: Forced expiratory flow volume in 1 s
FVC: Forced vital capacity
GINA: Global Initiative for Asthma
Hb A1c: Glycosylated hemoglobin type A1C
HDMs: House dust mites
IRB: International Review Board
kDa: Kilo daltons
L. lactis: *Lactococcus lactis*
P-value: Probability value
ROC curve: Receiver operating characteristic curve
SCORAD index: Scoring of Atopic Dermatitis index
SD: Standard deviation
SPSS Program: Statistical Package for the Social Sciences Program
TEC: Total eosinophil count
